# Cyto‐Genotoxic Assessment of Sulfoxaflor in *Allium cepa* Root Cells and DNA Docking Studies

**DOI:** 10.1002/jemt.24807

**Published:** 2025-01-17

**Authors:** Recep Liman, Muhammad Muddassir Ali, Erman Salih İstifli, İbrahim Hakkı Ciğerci, Ümran Tınaz, Sidal Kırlangıç, Nejla Altay, Yudum Yeltekin Uğur

**Affiliations:** ^1^ Molecular Biology and Genetics Department, Faculty of Engineering and Natural Sciences Uşak University Uşak Turkey; ^2^ Institute of Biochemistry and Biotechnology University of Veterinary and Animal Sciences Lahore Pakistan; ^3^ Cukurova University Faculty of Science and Literature, Department of Biology Adana Turkey; ^4^ Molecular Biology and Genetics Department, Faculty of Science and Literatures Afyon Kocatepe University Afyonkarahisar Turkey

**Keywords:** *Allium cepa* ana‐telophase assay, DNA damage, insecticide, Sulfoxaflor, toxicity

## Abstract

Sulfoxaflor (SFX) is an insecticide that is commonly used for the control of sap‐feeding insects. Since SFX is extensively applied globally, it has been implicated in the substantial induction of environmental toxicity. Therefore, in this study, *Allium cepa* roots have been employed to elucidate the potential cytogenotoxic effects of SFX in non‐target cells by examination of mitotic index (MI), chromosomal aberrations (CAs), and DNA damage. Physiological effects of SFX were evaluated by *A. cepa* root growth inhibition assay, while cytogenotoxic effects were assessed by *A. cepa* ana‐telophase and comet assay. Moreover, DNA binding affinity and binding mode of SFX were examined using molecular docking simulations to shed light on the genotoxic mechanism of action. The half maximal effective concentration (EC_50_) on the growth of *A. cepa* cells calculated for SFX was found as 500 mg/L. Moreover, dose‐ and time‐dependent decrease in MI, increase in CAs (disturbed ana‐telophase, chromosomal laggards, stickiness, and anaphase chromosome bridge) and DNA damage were observed by the exposure of *A. cepa* root tips to SFX after 24‐, 48‐, 72‐, and 96‐h treatment periods. A 6‐bp double‐stranded DNA structure containing two intercalation sites (PDB ID: 1Z3F) was used for docking studies. According to DNA docking results, SFX exhibited an energetically more favorable binding affinity with DNA (Δ*G* = −5.05 kcal/mol) compared with the experimental mutagen methyl methanesulfonate (MMS) (Δ*G* = −2.94 kcal/mol), and preferentially snugly fits into the minor groove of DNA possessing an intercalation gap, thus, providing valuable mechanistic data into the formation of chromosome aberrations and DNA fragmentation induced by this pesticide in *A. cepa.*

## Graphical abstract

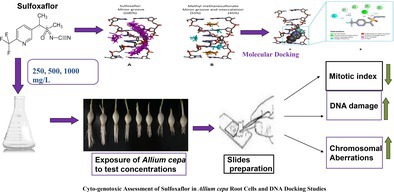

**Summary**

- Sulfoxaflor (SFX) significantly inhibited onion root growth and mitotic index.
- SFX exposure resulted in a dose‐ and time‐dependent increase in chromosomal aberrations.
- SFX induced DNA damage in *A. cepa* root tips.
- Molecular docking simulations revealed that SFX binds more strongly to DNA than MMS.
- These findings provide mechanistic insights into SFX‐induced cytogenotoxicity in non‐target organisms.

## 1 Introduction

Insecticide resistance in agricultural settings stands as one of the most significant challenges nowadays. Many insects such as *Aphis gossypii* , *Bemisia tabaci* have already developed resistance against a broad range of insecticides (Sparks et al. 2013). Sulfoximines (Group 4C), a new class of insecticides based on the sulfoximine moiety, has been developed to overcome insecticide resistance. A compound of this class named Sulfoxaflor (SFX) is now used as a rational alternative to neonicotinoid insecticides, in more than 80 countries in the world (Siviter et al. 2020). SFX is effective against sap‐feeding insect pests like aphids, bugs, and whiteflies (Ma et al. 2019). SFX is used as a spray solution and there has been a variation in its residual levels from one crop to another (Kyriakopoulou et al. 2017). As its mechanism of action, SFX binds to nicotinic acetylcholine receptors (nAChRs) of insects as a competitive modulator/agonist (Zhu, Loso, and Watson 2011). The formation of SFX‐nAChR complex, in turn, leads to an increase in the rate of neurotransmission in the central nervous system (CNS) which results in the observed deleterious effects such as muscle tremors followed by paralysis and eventually the death of insects (Ihara and Matsuda 2018).

SFX is a group that shows some similarities in the mode of action as the neonicotinoids (Group 4A) (Chung et al. 2017; Skouras et al. 2023), but with differences in its sensitivity to enzymes associated with resistance to some insecticides as well as in the interaction with its target receptor due to its unique molecular structure (especially the sulfoximine moiety) (Watson et al. 2021). Generally, this compound is effective against insects such as brown plant hopper and white fly which are resistant to other insecticides like imidacloprid (Babcock et al. 2011; Sparks et al. 2013; Cheng et al. 2018). The structure of SFX is stable to the extent that it is not altered even in the presence of monoxygenase enzymes resulting in a lack of cross‐resistance (Cheng et al. 2018). SFX is degraded into two metabolites, that is, X11719474 and X1172061 which in turn cause toxic effects in rats (Chung et al. 2017). According to EU pesticides peer reviews under Regulation (EC) No. 1107/2009 minimum non‐toxic daily intake value of SFX is 0.04 mg/kg per day, while the acute reference dose is 0.25 mg/kg (EFSA (European Food Safety Authority) 2014).

Due to the systemic nature of SFX, following application, small amounts of SFX are identified in nectar and pollen grains of plants, which, in turn, pose deleterious health effects to human beings and animals (Siviter, Brown, and Leadbeater 2018). In rodents, long‐term oral exposure to SFX leads to hepatic tumor formation (LeBaron et al. 2013). In rats, this exposure leads to various abnormalities of the testes, seminiferous tubules, and a reduction in a number of sperms (EFSA (European Food Safety Authority) 2014). Moreover, in earthworms, this insecticide causes the generation of reactive oxygen species (ROS) and lipid peroxidation (Zhang et al. 2020).

The toxic effects of various chemical compounds can be assessed using a range of assays. Among these, the *A. cepa* and the comet assay, used in combination, are the most commonly employed methods in genotoxicity testing (Cabuga Jr. 2017). Using the *A. cepa* assay, the cytogenotoxicity, clastogenicity, and aneugenicity of toxic compounds can be reliably evaluated (Ayhan et al. 2024; Banti and Hadjikakou 2019; Sheikh, Patowary, and Laskar 2020; Liman et al. 2021, 2022). *A. cepa* ana‐telophase test is fast, cost‐effective, easy‐to‐perform, and produces relevant results (Bakadir et al. 2016; Bonciu et al. 2018). Comet assay or Single Cell Gel Electrophoresis assay is used to assess the level of DNA damage (in the form of DNA fragmentation) induced by various mutagens, chemicals, nanoparticles, fungicides, and pesticides (Glei, Schneider, and Schlormann 2016; Moller 2018; Ali et al. 2022b; Ambreen et al. 2023). For three decades, this assay has been widely used, as it is a unique, simple, and sensitive technique to study the genotoxicity of chemical compounds at the genome level (Silveira et al. 2017; Pellegri, Gorbi, and Buschini 2020; Ali and Ciğerci 2019; Liman and Ozkan 2019; Karaismailoğlu 2022; Turan et al. 2023).

Molecular docking studies have been used to elucidate the reactive nature of small molecules that have the potential to cause toxicity in cells by binding against target macromolecules such as proteins, DNA, or RNA (Alesawy et al. 2021; Daina, Michielin, and Zoete 2017; Alshehri et al. 2023). Moreover, unknown binding sites of ligands that target specific receptors can be also identified through this molecular simulation technique (Gilad and Senderowitz 2014; Wang and Zhu 2016; Torres et al. 2019; Bouhadi et al. 2024). *In silico* molecular docking, technique is commonly used in structure‐based drug design and elaboration of the mechanism of action of various genotoxic substances (Liman et al. 2021; Wang and Zhu 2016; Torres et al. 2019). Recently, with the number of various synthetic small molecules in online databases increasing rapidly, molecular docking method has become an important bioinformatics method in the elucidation of intermolecular interactions between these different synthetic structures and their off‐target receptors (a protein or nucleic acid that the ligand binds nonspecifically, which could lead to adverse or side effects in biological systems), thus, providing a solid insight into the underlying mechanism of the negative biological response which is an undesirable or harmful effect on the organism (i.e., genotoxicity) (Ricci and Netz 2009). In this context, although the DNA molecule is an important macromolecular target of synthetic small molecules (i.e., pesticides, antibiotics, and antitumor drugs), disappointingly, the DNA docking approach is rarely applied in the investigation of the molecular mechanisms of genotoxic response elicited by these molecules (Gilad and Senderowitz 2014; Ricci and Netz 2009).

Therefore, this study aimed to investigate root growth inhibition, mitotic index (MI), chromosomal aberrations (CAs), and DNA damage induced by the pesticide SFX in *A. cepa* root meristematic cells. Moreover, the molecular docking method was also used to probe the molecular mechanisms of CAs and DNA damage induced by SFX, thus, the DNA‐binding mode, DNA‐binding affinity, and resulting intermolecular interactions of SFX were elucidated at the molecular level, providing mechanistic insights into the cytogenotoxic effects induced by this sulfoximine pesticide in *A. cepa* roots.

## 2 Materials and Methods

### 2.1 Root Growth Inhibition Assay

To assess the physiological effects of SFX, a root growth inhibition test was performed with some modifications (Ali et al. 2022b; Liman et al. 2022). Isoclast active, a 50% SFX suspension concentrated formulation produced by Dow AgroSciences (USA), was sourced from a local pesticide retailer in Antalya, Turkey. Organically grown onions (25–30 mm diameter) used in experiments were purchased from the local market. Dead roots of all onions were removed and then they were exposed to various concentrations of SFX (100, 200, 300, 400, 500, 600, 700, and 800 mg/L), as well as to distilled water as a negative control, for 96‐h in the test tubes at room temperature. After the exposure period, the mean root length from five onions (10 roots from one bulb) per each treatment was measured and then the value of half maximal effective concentration (EC_50_) was calculated.

### 2.2 Allium Ana‐Telophase Test

As stated by Rank and Nielsen (1994), the Allium ana‐telophase test was carried out with a few minor modifications. *A. cepa* bulbs were exposed to distilled water for 48‐h in test tubes and then freshly grown roots were exposed to various concentrations (250, 500, and 1000 mg/L) of SFX. Test concentrations along with positive control (10 mg/L MMS) and negative control (distilled water) were then kept in the dark at room temperature for 24‐, 48‐, 72‐, and 96‐h. Five to eight of the onion's root tips, each measuring ~1 cm in length, were taken from three onions for every concentration. Root tips were kept in Carnoy fixative (3:1; ethanol: glacial acetic acid) at 4°C for 24‐h. After that, root tips were washed with distilled water and kept in 70% alcohol at 4°C. Before experimentation, roots were washed with distilled water and then kept in 1 N HCl solution for 8 min at 60°C. Schiff's reagent was used to stain the root tips for 20–30 min. A root tip of 1–2 mm was cut using a sterile fresh razor blade and then slides were prepared using the squash technique. After the preparation of slides, they were examined under the light microscope (Nikon Eclipse Ci‐L, Japan) at 400x magnification. MI, phase indices, and CAs were calculated using the following formulas:

$$\mathrm{MI}\left(\%\right)=\frac{\mathrm{No}.\text{of cells in cell division}}{\mathrm{No}.\text{of total cells observed}}\times 100$$

$$\text{Phase Index}\left(\%\right)=\frac{\mathrm{No}.\text{of cells in particular phase}}{\text{Total}\;\mathrm{No}.\text{of cells in cell division}}\times 100$$

$$\mathrm{CA}\left(\%\right)=\frac{\text{Total}\;\mathrm{ana}-\text{telophase anomalies}}{100\;\mathrm{ana}-\text{telophase cells}}\times 100$$

A total of 5000–5500 cells from five slides (1000–1100 cells per slide) of each concentration were counted to find MI and phase indices. Similarly, a total of 500 ana‐telophase cells (100 ana‐telophase cells per slide) for each concentration were counted to calculate the frequency of CAs.

### 2.3 Comet Assay

**FIGURE 1:**
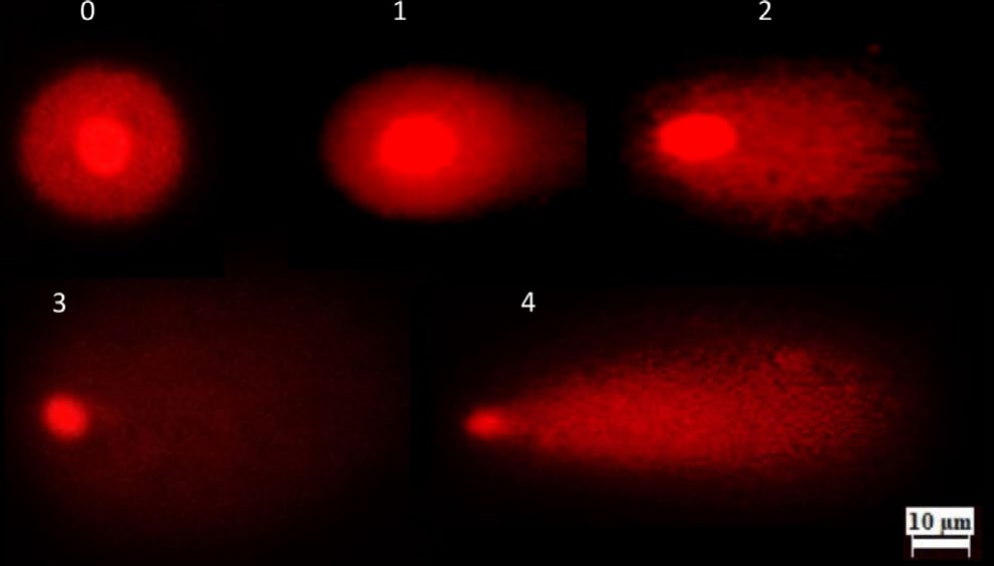
Comet scores of DNA damage induced by SFX in *A. cepa* roots at 400 × magnification. 0—no damage (< 5%), 1—minor damage (5%–20%), 2—moderate damage (20%–40%), 3—severe damage (40%–85%), 4—complete DNA damage (> 85%).

Comet assay was carried out to assess the DNA damage on root tips of *A. cepa* after exposure to various SFX concentrations (250, 500, and 1000 mg/L) along with positive control (10 mg/L MMS) and negative control (distilled water) groups according to Ali et al. (2022a) and Liman et al. (2022) with some minor modifications. Three bulbs were utilized for each treatment and control group under the identical conditions outlined in the Allium ana‐telophase test. The treated root tips (30 for each treatment) about 0.5 mm from their ends were carefully cut. Next, the root tips were chopped with a new razor blade in 600 μL of cold nuclear isolation buffer (4 mM MgCl_2_‐6H_2_O, 0.5% w/v Triton X‐100, 0.2 M Tris, and pH 7.5) and filtered using a nylon filter with 60‐μm mesh to isolate nuclei. Following a 7‐min centrifugation at 1200 rpm and 4°C, the supernatant was discarded. A 50 μL suspension of nuclei was combined with 50 μL of 1.5% low‐melting point agarose in Tris–HCl buffer and gently spread onto slides that had been pre‐coated with a layer of 1% normal‐melting point agarose. After that slides were kept on ice slabs for 5 min. Then they were kept in an ice‐cold buffer solution (NaOH, EDTA; pH > 13) for 20 min. Three slides per treatment were prepared and these slides were then subjected to electrophoresis at 25 V and 300 mA for 20 min. After removing slides from the electrophoresis buffer, slides were neutralized using neutralization buffer thrice for 5 min and stained with 80 μL of 20 μg/mL ethidium bromide solution. Using a BAB fluorescence microscope (TAM‐F, Turkey), 50 randomly chosen comets per slide (150 comets per treatment) were analyzed at 200x magnification to determine the amount of DNA damage expressed as an arbitrary unit. The comets were categorized into five damage levels based on head integrity and tail length, with values ranging from zero to four, as outlined by Kocyigit et al. (2005) and illustrated in Figure 1. Where score 0 represents no damage (< 5%), 1 for minor damage (5%–20%), 2 for moderate damage (20%–40%), 3 for severe damage (40%–85%) and 4 represents complete DNA damage (> 85%). The following formula was used to evaluate the DNA damage induced by different concentrations of SFX.

$$\text{Arbitrary damage}=\sum_{i=0}^{4}\mathrm{Ni}\times i$$

In the above equation, N_*i*_ represents the number of cells in a particular degree of damage in the 5‐point scale; *i* represents the degree of damage (0, 1, 2, 3, and 4).

### 2.4 DNA Docking Studies of SFX

#### 2.4.1 Nucleic Acid/Ligand Retrieval and Preparation

**FIGURE 2:**
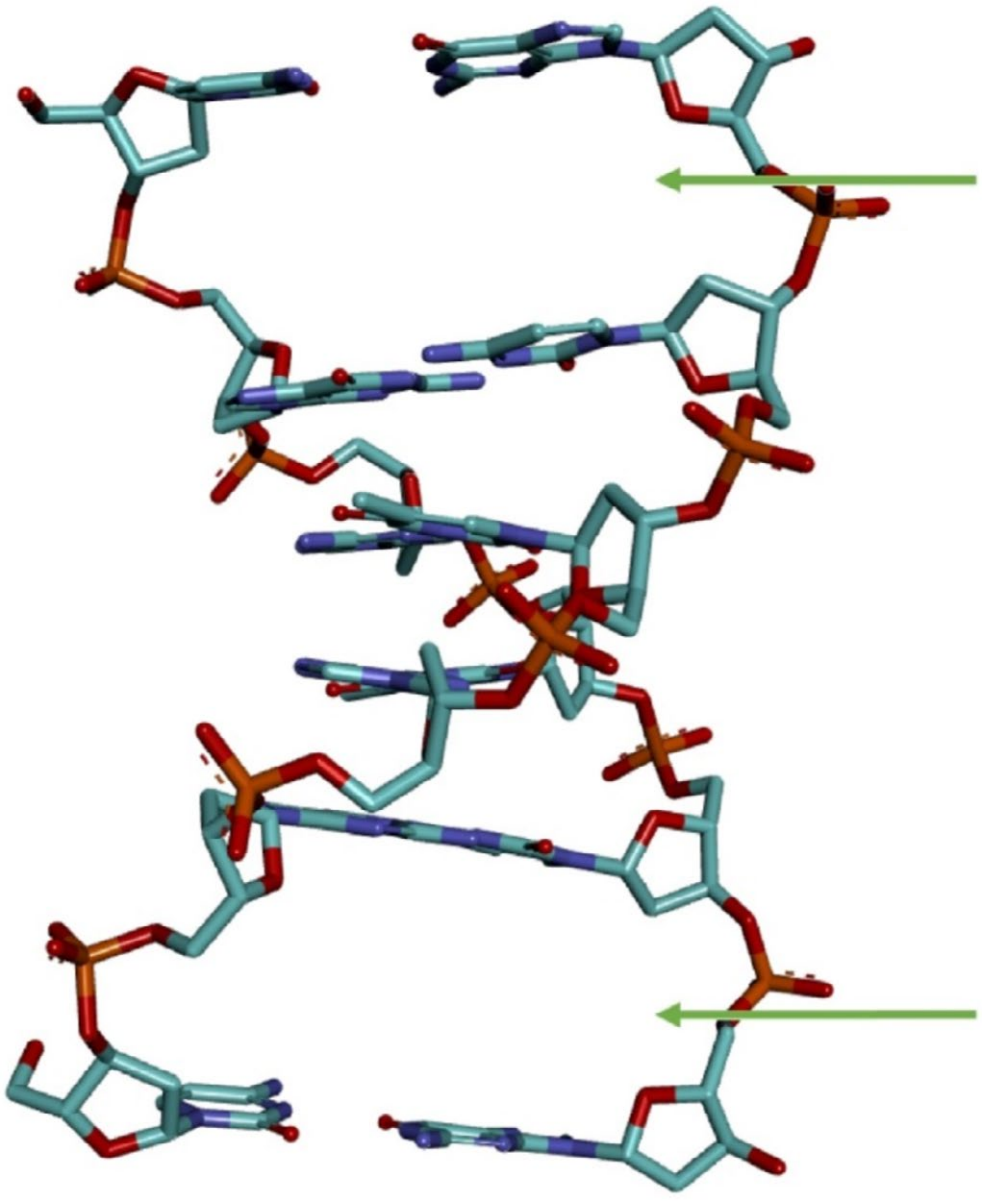
Three‐dimensional structure of the 6‐base‐pair DNA oligonucleotide (in stick mode) with intercalation gaps (PDB ID: 1Z3F) used in the docking experiments. Docking studies involving DNA with intercalation sites provide a valuable approach for elucidating alternative DNA binding mechanisms of chemical entities with unknown binding modes. The two intercalation gaps are indicated with green arrows.

Molecular docking was performed to probe the molecular mechanisms of DNA damage and chromosomal abnormalities induced by SFX, thus, the DNA‐binding mode, DNA‐binding affinity, and resulting intermolecular interactions of SFX were elucidated. In a previous spectroscopy and docking study, it has been reported that acetamiprid (the neonicotinoid insecticide), a structurally close congener of SFX, binds the DNA molecule in a partial intercalative mode (Zhang et al. 2013). Therefore, in order not to rule out the possible DNA‐intercalative binding mode of SFX, our docking studies were conducted with a unique crystallographic DNA conformation carrying a natural intercalation gap (Figure 2). Molecular docking considering the DNA conformation with an intercalation gap has been shown to be the most appropriate method for the elucidation of possible intercalative binding mode of various ligands (Ricci and Netz 2009). Therefore, in docking experiments performed with SFX, a DNA conformation possessing an intercalation gap (PDB ID: 1Z3F, resolution: 1.50 Å) downloaded from the RCSB PDB database was used as the target receptor. In addition, MMS, the experimental positive control of the study, was used as the reference compound in docking experiments, and the results obtained from docking with SFX were compared with the docking results of MMS.

Before molecular docking, water molecules and other non‐interacting ions (i.e., magnesium) surrounding the DNA receptor were removed using the Discovery Studio Visualizer v16 program (Biovia 2016). On the other hand, the crystal structures of SFX and MMS (ligands) were downloaded in structure‐data format (sdf) from PubChem database (https://pubchem.ncbi.nlm.nih.gov/). They were saved in their lowest energy conformation (mol2 format) by optimizing using MMFF94 force field with the steepest descent algorithm in the Avogadro program (Hanwell et al. 2012). Before docking, these receptor and ligand structures (pdb and mol2 files, respectively) were converted to pdbqt format using AutoDock Tools 1.5.6 (Morris et al. 2009).

#### 2.4.2 Molecular Docking

In this study, the latest version of AutoDock Vina (1.2.3) was used to perform docking of SFX against the DNA receptor carrying intercalation gap (Eberhardt et al. 2021). To perform this task, AutoDock Vina utilizes a gradient‐based local search genetic algorithm to find optimal poses of ligands within the receptor binding sites (Sarkar et al. 2024).

In docking studies performed using the DNA with intercalation gap, polar hydrogens in receptor and ligands were kept, however, non‐polar hydrogens were merged using AutoDock Tools 1.5.6. Kollman charges were added to the receptor molecule, whereas the Gasteiger charges were assigned to the ligands. During the docking calculations, the rotatable bonds of the ligands were chosen to free rotation, whereas the receptor was kept in a rigid configuration. A grid box size of 70 × 68 × 80 Å points (center x = 2.109; center y = 15.509; center z = 37.148) for DNA carrying intercalation gap was adjusted. Since the DNA molecule does not have a predetermined “*active site*” like proteins, the x, y, and z coordinates of the grid box were determined to allow SFX and MMS to interact with the entire surface (the minor and major grooves) of the DNA conformation. Also, the binding energy value obtained as a result of docking of MMS with the DNA conformation was used as a positive control value for comparison in SFX‐DNA docking.

The “*number of docking runs*” was set as “20” and the “*exhaustiveness*” was set to “100” in the dockings of SFX (as well as the MMS) against the DNA target. Following the docking calculations, all potential binding modes of SFX and MMS were clustered through AutoDock Vina 1.2.3 and were ranked based on the lowest binding free energy (Δ*G*; kcal/mol) of the ligands against the target receptor. The top‐ranked conformations of SFX (as well as the MMS) among different poses against the DNA target were visualized and analyzed using the Discovery Studio Visualizer v16 program (Biovia 2016). In addition, all binding conformations (20 conformations for each ligand) obtained from docking of SFX and MMS with the DNA target and the clusters they formed are given in a separate figure to understand the most likely DNA‐binding mode of both ligands.

#### 2.4.3 Statistical Analysis

Results were represented as mean ± standard deviation. One‐way ANOVA along with Duncan and Pearson correlation tests were performed to make comparison analysis among groups and bivariate correlations of groups, respectively, at *p* ≤ 0.05 and *p* = 0.01 using IBM SPSS version 23 for Windows.

## 3 Results

### 3.1 *A. cepa* Root Growth Inhibition Test

**FIGURE 3:**
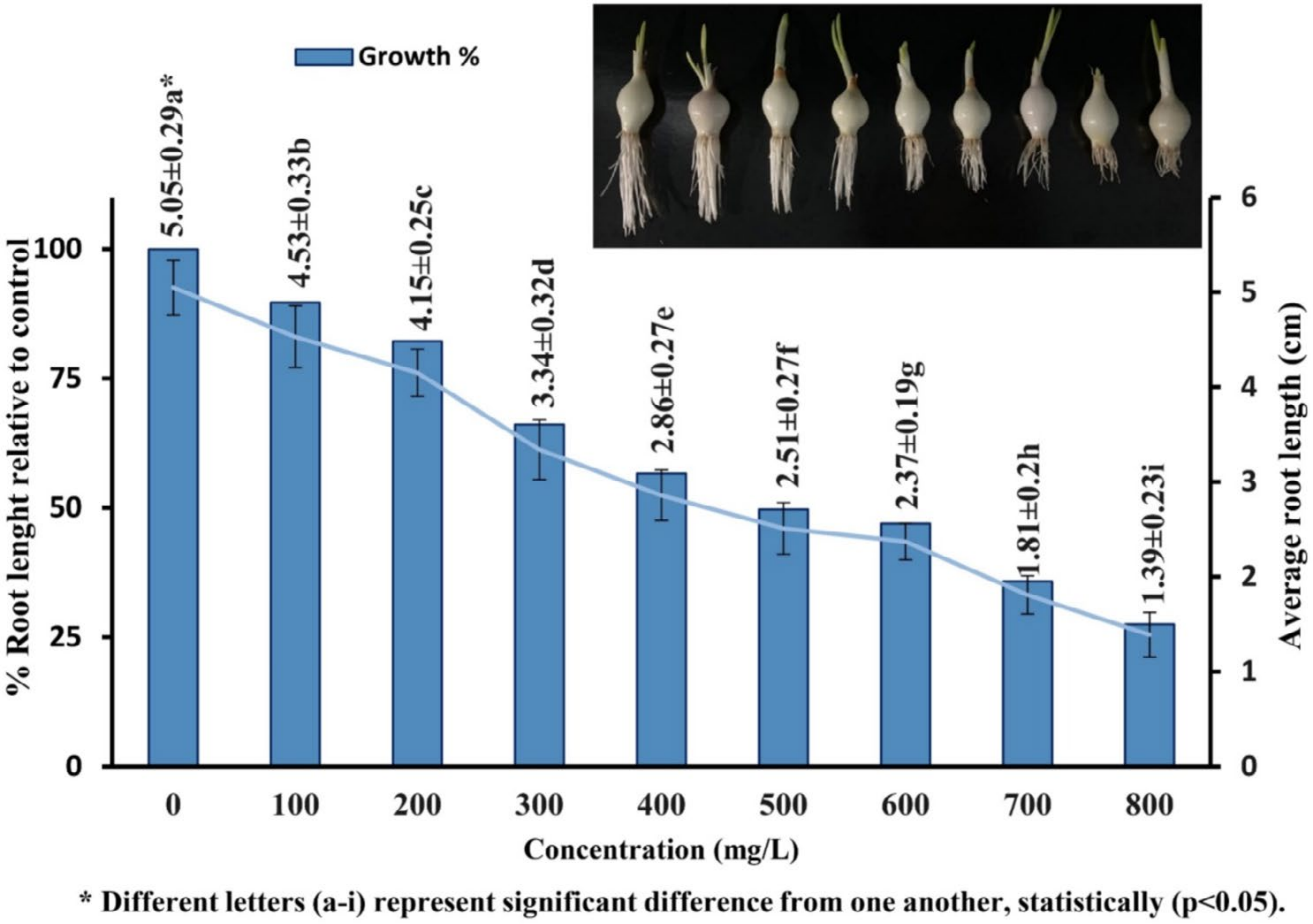
Findings of the *A. cepa* root growth inhibition test after exposure to different SFX concentrations at 96 h.

The EC_50_ of SFX was found to be 500 mg/L (50.3%) by *A. cepa* root growth inhibition test (Figure 3). The results of the root growth inhibition assay showed statistically significant (*p* ≤ 0.05) dose‐dependent inhibition (*r* = −0.958) due to SFX exposure. The highest value (1.39 ± 0.23 cm) of root growth inhibition was observed at 800 mg/L concentration of SFX. On the other hand, the lowest value (4.15 ± 0.25 cm) of root growth inhibition after treatment with SFX was observed for the concentration of 100 mg/L.

### 3.2 Cytogenotoxicity of SFX

Findings of mitotic and phase indices after treatment with various concentrations of SFX at different time intervals (24‐, 48‐, 72‐, and 96‐h) are shown in Table 1. A significant decrease (*p* ≤ 0.05) in the values of MI was observed due to SFX treatment, in a dose‐dependent (*r* = −0.911 for 250 mg/L, *r* = −0.961 for 500 mg/L, and *r* = −0.951 for 1000 mg/L) and time‐dependent (for 24‐h *r* = −0.934, for 48‐h *r* = −0.880, and for 96‐h *r* = −0.773) manner compared with the negative control. Compared with the control group, SFX exposure considerably decreased the prophase indices statistically significantly, whereas telophase indices increased significantly except for the 48‐h application.

**TABLE 1:** Effect of SFX on mitotic and phase index in *A. cepa* roots with respect to various concentrations and time intervals.

Concentration (mg/L)	TCC	CMS	MI ± SD*	Phase index (%) ± SD*			
				Prophase	Metaphase	Anaphase	Telophase
Control‐24 h	5163	3701	71.69 ± 1.22a	92.52 ± 0.54a	1.27 ± 0.15a	1.46 ± 0.14a	4.75 ± 0.49a
MMS‐10	5291	3149	59.52 ± 1.40b	87.36 ± 0.57b	2.29 ± 0.17b	2.99 ± 0.21b	7.37 ± 0.28b
250	5357	3547	66.22 ± 0.29c	90.21 ± 0.37c	1.61 ± 0.09c	1.72 ± 0.08a	6.47 ± 0.29c
500	5337	3418	64.05 ± 0.65d	87.83 ± 0.26b	2.34 ± 0.19b	2.75 ± 0.20b	7.08 ± 0.26b
1000	5461	3438	62.96 ± 0.47d	87.63 ± 0.50b	2.62 ± 0.16d	2.74 ± 0.36b	7.02 ± 0.26b
Control‐48 h	5106	3557	69.66 ± 0.75a	89.85 ± 0.70a	1.52 ± 0.24a	1.54 ± 0.18a	7.08 ± 0.46ab
MMS‐10	5432	3240	59.65 ± 0.77b	87.50 ± 0.40b	2.25 ± 0.20b	3.00 ± 0.25b	7.26 ± 0.55b
250	5200	3321	63.87 ± 0.55c	90.15 ± 0.64a	1.57 ± 0.23a	1.72 ± 0.18a	6.56 ± 0.35a
500	5226	3288	62.92 ± 0.48d	90.02 ± 0.51a	1.49 ± 0.17a	1.70 ± 0.14a	6.79 ± 0.33ab
1000	5203	3231	62.10 ± 0.17e	90.00 ± 0.10a	1.33 ± 0.16a	1.48 ± 0.12a	7.19 ± 0.32b
Control‐72 h	5147	3551	68.99 ± 0.63a	90.87 ± 0.43a	1.49 ± 0.08a	1.52 ± 0.11a	6.11 ± 0.40a
MMS‐10	5382	3094	57.50 ± 1.63b	86.94 ± 0.43b	2.46 ± 0.13b	2.78 ± 0.21b	7.82 ± 0.27b
250	5172	3144	60.80 ± 1.01c	89.18 ± 0.26c	1.40 ± 0.15a	1.46 ± 0.09a	7.95 ± 0.08b
500	5293	3216	60.76 ± 0.58c	89.07 ± 0.60c	1.40 ± 0.17a	1.43 ± 0.16a	8.09 ± 0.40bc
1000	5232	3140	60.01 ± 0.52c	88.76 ± 0.43c	1.40 ± 0.15a	1.43 ± 0.15a	8.41 ± 0.29c
Control‐96 h	5182	3540	68.32 ± 1.00a	90.98 ± 0.17a	1.36 ± 0.16a	1.38 ± 0.06a	6.27 ± 0.20a
MMS‐10	5283	2925	55.37 ± 0.54b	86.29 ± 0.34b	2.57 ± 0.32b	2.87 ± 0.16b	8.28 ± 0.23b
250	5214	3111	59.68 ± 1.76c	88.49 ± 0.27c	1.48 ± 0.16a	1.45 ± 0.14a	8.59 ± 0.20c
500	5159	3055	59.23 ± 048c	88.22 ± 0.09c	1.41 ± 0.10a	1.31 ± 0.16a	9.07 ± 0.03d
1000	5294	3010	56.86 ± 0.75d	87.04 ± 0.48d	1.43 ± 0.18a	1.50 ± 0.15a	10.04 ± 0.32e

Abbreviation: CMS: Cells in Mitotic Stage, MI: Mitotic Index, SD: Standard Deviation, TCC: Total Cell.Counted.

* Different letters in the same columns for each treatment time are significantly different (*p* < 0.05).

**FIGURE 4:**
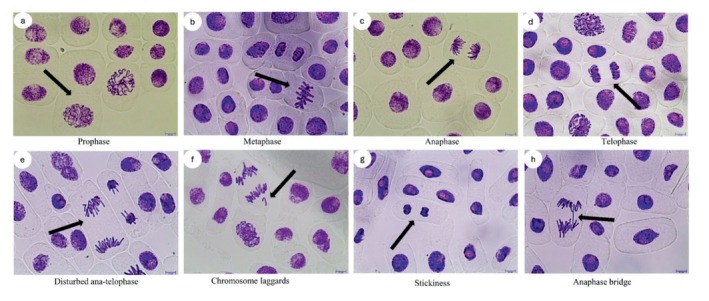
Normal mitotic phases and anomalies induced by SFX in root tips of *A. cepa*. (a): Prophase, (b): Metaphase, (c): Anaphase, (d): Telophase, (e): Disturbed anaphase‐telophase, (f): Chromosome laggards, (g): Stickiness, (h): Anaphase chromosome bridge, Scale bars = 10 μm with 400 x magnification.

**FIGURE 5:**
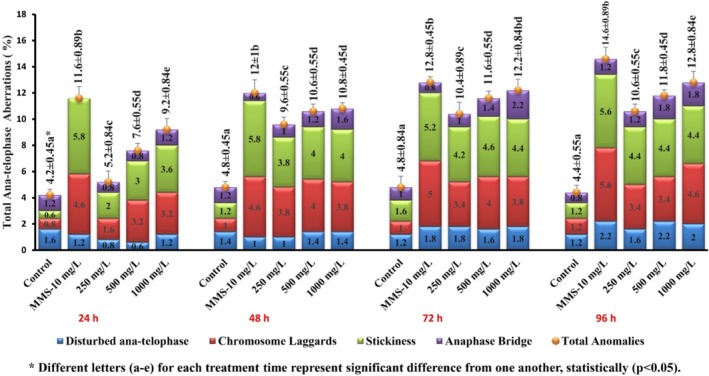
CAs observed induced by SFX observed in *A. cepa* ana‐telophase cells at various concentrations and time intervals.

The type of CAs observed due to SFX treatment were disturbed ana‐telophase, chromosomal laggards, stickiness, and anaphase chromosome bridge (Figure 4). The increased number of CAs was observed when *A. cepa* cells were exposed to various SFX (250, 500, and 1000 mg/L) concentrations (Figure 5). The highest frequency (12.08 ± 0.84) of CA upon SFX treatment was observed in the group treated with 1000 mg/L concentration of SFX at 96‐h with TCC of 5294 and MI of 56.86 ± 0.75. The observed increase in these CAs was associated with an increase in the concentrations of SFX (*r* = 0.830 for 250 mg/L, *r* = 0.872 for 500 mg/L, and *r* = 0.883 for 1000 mg/L) as well as the duration of exposure (for 24‐h r = 0.919, for 48‐h *r* = 0.701, for 72‐h *r* = 0.721, and for 96‐h *r* = 0.845), when compared with negative control.

**FIGURE 6:**
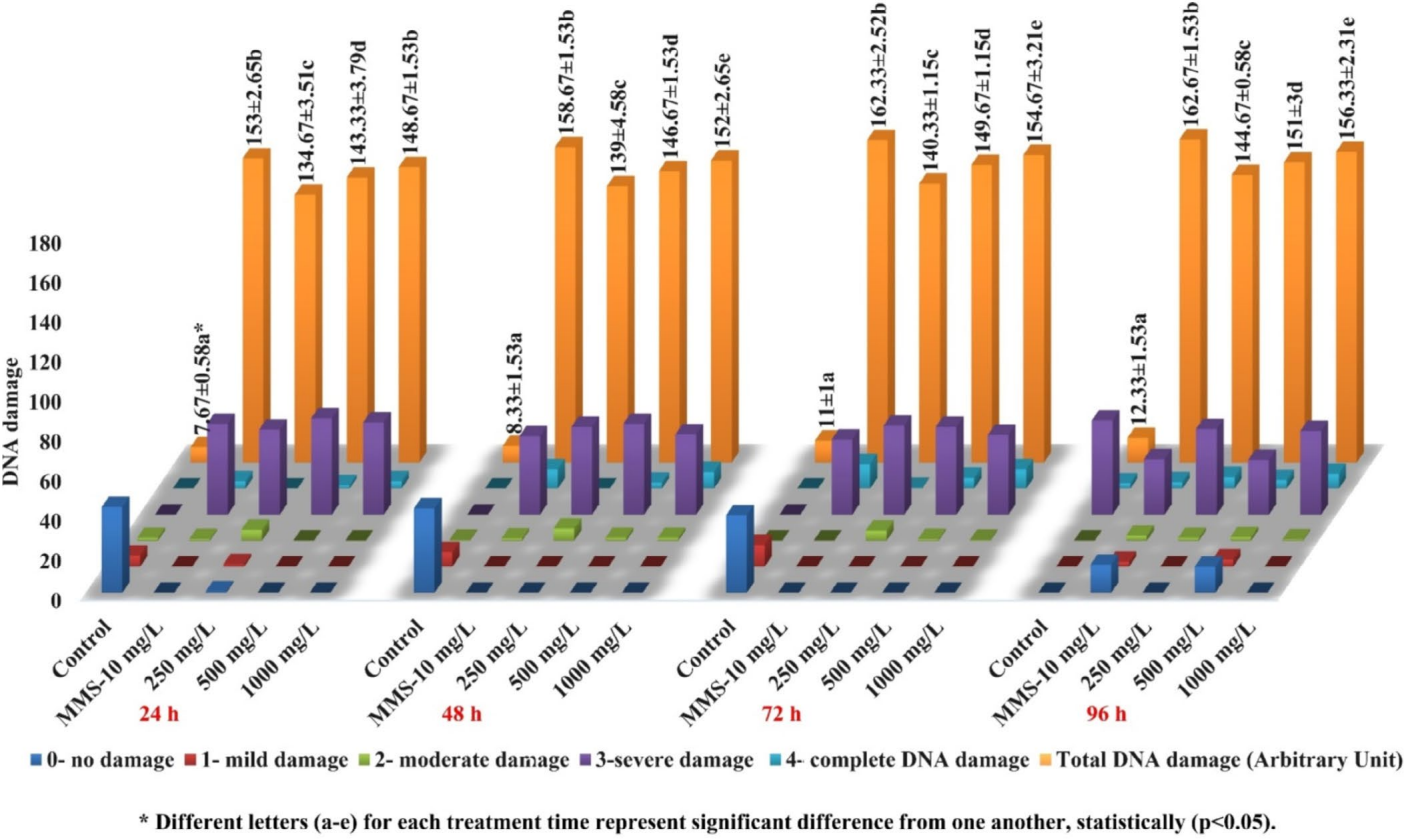
DNA damage findings in the nuclei of *A. cepa* root meristems after SFX exposure.

DNA damage due to SFX treatment was increased in a time‐ (for 24‐h *r* = 0.907, for 48‐h *r* = 0.894, for 72‐h *r* = 0.947 and for 96‐h *r* = 0.934) and concentration‐ (*r* = 0.813 for 250 mg/L, *r* = 0.799 for 500 mg/L, and *r* = 0.809 for 1000 mg/L) dependent manner. The highest value of DNA damage (156.33 ± 2.31) was observed after 96‐h exposure to SFX (Figure 6).

### 3.3 DNA Docking Studies of SFX

**FIGURE 7:**
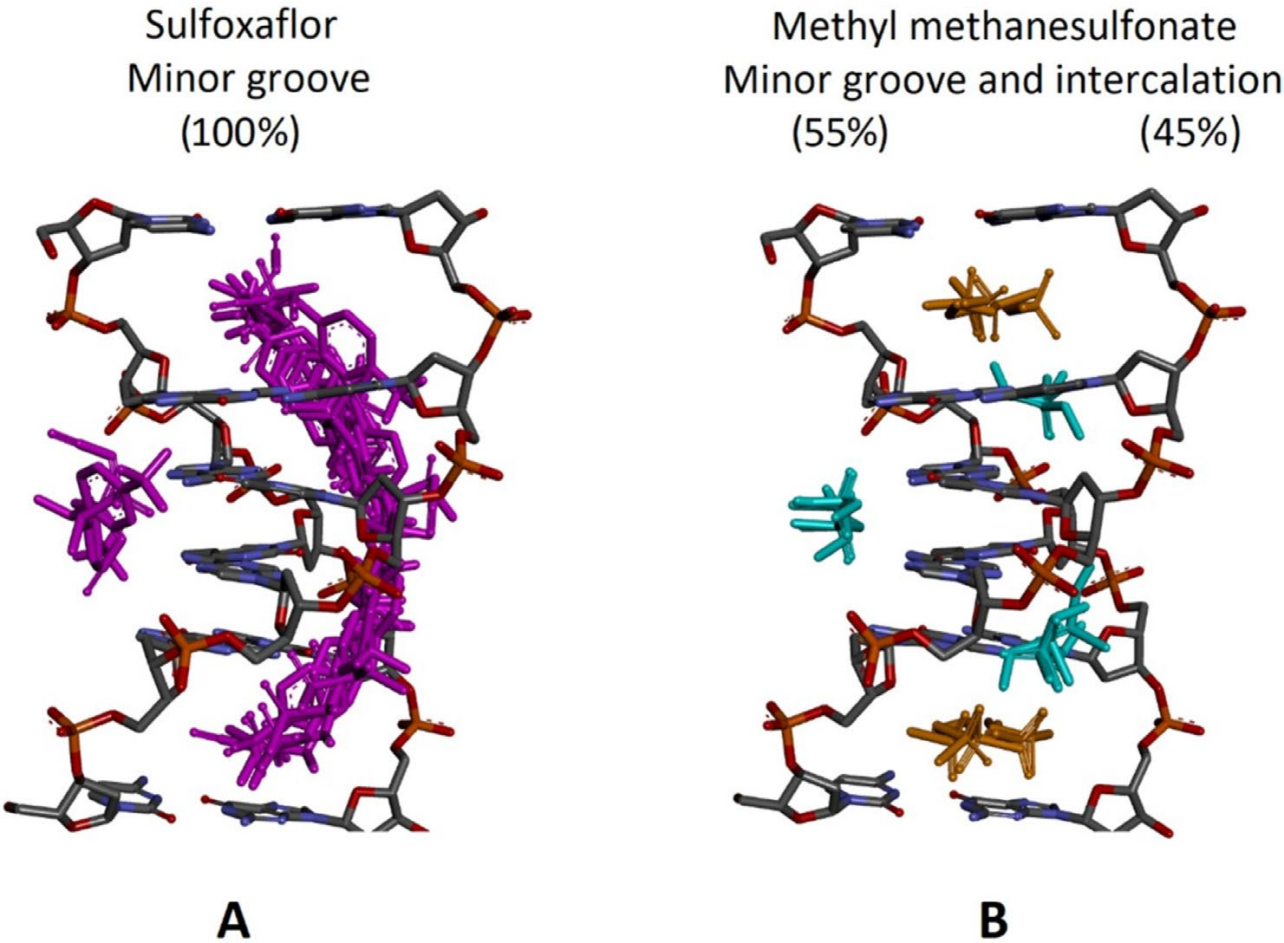
Conformation clusters calculated by AutoDock Vina as a result of docking of SFX and MMS with the target DNA carrying intercalation gap (PDB ID: 1Z3F) (20 docking runs in total for SFX and MMS) and individual binding poses that populate these clusters. Note that while SFX (A, ligand in magenta color) interacts consistently with DNA minor groove, MMS interaction with DNA (B, orange, and cyan color for two different binding modes) is mixed type: Both intercalation and minor groove recognition. Ligands and receptors are depicted in stick mode. The figure was prepared using DS Studio Visualizer v16. groove recognition.

To investigate the DNA‐binding mode, DNA‐binding affinity, and resulting intermolecular interactions of SFX, docking studies were performed using a DNA conformation possessing intercalation gap. This methodology, using a DNA conformation with intercalation gap, is useful for the accurate prediction of binding modes of various small molecule ligands with DNA. Moreover, docking studies were also performed for the positive control compound MMS against DNA, to rationalize the results obtained from docking of SFX. Interestingly, SFX exhibited a consistent preference for minor groove binding (100%) (Figure 7A), with a mean binding free energy (Δ*G*) of −5.05 kcal/mol (Table 2).

**TABLE 2:** Docking binding mode, mean binding free energy, and binding interactions calculated from interactions of SFX and positive control compound (MMS) with target DNA conformation.

Ligand	Binding mode	%	Mean binding free energy* (Δ*G*: kcal/mol)	Binding interactions of the top‐ranked conformations^Ω^				
				H‐Bond	van der Waals	Hydrophobic	Halogen	Other (π‐sulfur)
						π–π/π–alkyl/pi‐sigma		
SFX	Minor groove	100	−5.05	G2, A3	T4, C5, G6	G2, A3	A3, T4	—
MMS	Minor groove	55	−2.90	G2	A3, T4	—	—	G2
	Intercalation	45	−2.94	C1, G6	—	G2	—	—

* Mean binding free energy (kcal/mol) is given as the average of 20 total docking runs.

^**Ω**^ Top‐ranked conformation (conformation with the most negative binding free energy) was taken into account in the intermolecular interactions of SFX and MMS with DNA.

The binding mode of the positive control ligand, MMS, is mixed type: both minor groove recognition (55%) and intercalation (45%) (Figure 7B). Despite the mean binding free energies of these two binding modes of MMS are very close to each other (Δ*G* = ‐2.90 kcal/mol for minor groove recognition; Δ*G* = ‐2.94 kcal/mol for intercalation, Table 2), it was determined that the intercalation mode of MMS is energetically more favorable (Δ*G* = ‐2.94 kcal/mol) (Table 2). In this binding mode, MMS is stacked between the C1 and G2 bases of DNA and forms three H‐bonds with G6 and C1, as well as two hydrophobic pi‐sigma contacts with G2 (image not shown).

**FIGURE 8:**
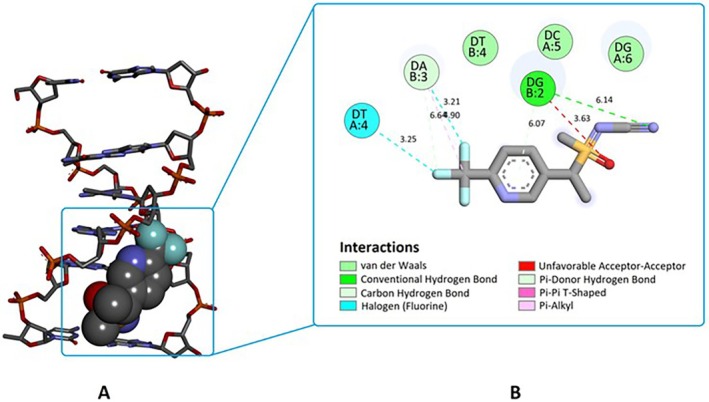
Binding mode of SFX with DNA carrying intercalation gap (PDB ID: 1Z3F). A. 3D image of SFX's minor groove recognition mode (DNA receptor in stick mode and SFX in CPK mode). Note that SFX fits snugly into the minor groove of the DNA structure. B. 2D interaction diagram of SFX with the DNA fragment. Green and light green dashed lines represent H‐bonds, cyan dashed lines represent halogen bonds, whereas violet and light violet dashed lines represent hydrophobic contacts. Resulting bond lengths (Å) are given next to each dashed line. The figure was prepared using DS Studio Visualizer v16.

The top‐ranked minor groove binding mode of SFX is depicted in Figure 8A, which is shown in CPK mode, and the DNA fragment in stick mode. The highly negatively charged trifluoromethyl group of SFX forms two halogen bonds with the DNA bases A3 and T4, as well as a hydrophobic pi‐alkyl and an H‐bond interaction also with A3 (Figure 8B). The pyridine moiety of SFX is aligned into the minor groove of the helix, showing an H‐bond and a hydrophobic pi‐pi T‐shaped interaction with G2 (Figure 8B). Finally, the N atom of the cyano group of SFX forms an H‐bond interaction with G2 (Figure 8B). It is also worth noting that in this most favorable conformation of SFX in the DNA minor groove, the cyano group of SFX slightly bends inward the minor groove and shows a partial intercalation. This could be attributed to the co‐operative effect of the flexibility of methylene bridge, sulfoximine, and cyano groups, and evokes a binding mode like that of acetamiprid.

Considering the docking binding affinities of SFX and MMS against DNA (Table 2), it was determined that the binding interaction of SFX (Δ*G* = ‐5.05 kcal/mol) with DNA was quite strong compared with the known DNA‐damaging mutagen MMS (Δ*G* = ‐2.94 kcal/mol), showing the DNA damage‐inducing capacity of SFX.

## 4 Discussion

In the current study, dose‐dependent root growth inhibition and significant decreases in the MI were observed upon SFX treatment compared with the negative control (Figure 3, Table 1). Our findings are consistent with the previous experimental results which imply inactivation of enzymes and disturbance in cell division which occur in apical meristems when roots are exposed to toxic chemicals (Fusconi et al. 2006; Aydın and Liman 2020). Reduction in MI occurs when the G_1_ phase is blocked (El‐Ghamery, El‐Nahas, and Mansour 2000) or when the DNA replication process is perturbed during the S‐phase of the cell cycle (Gupta et al. 2018). However, there is another mechanism noted in which because of mitotic stress *chfr* checkpoint interrupts entry into the metaphase stage (Bhat, Singh, and Vig 2015; Alaguprathana et al. 2022). SFX treatment alters the distribution of cells across the four mitotic phases. A reduction in the prophase index indicates that SFX disrupts the initiation of mitosis by inhibiting the transition of interphase cells into prophase (Soliman and Ghoneam 2004). Conversely, the increased metaphase index may result from SFX's interference with spindle function, leading to cell cycle arrest at this stage (Singh and Roy 2017). This spindle dysfunction also impairs the metaphase‐to‐anaphase transition, causing a decline in the anaphase index. An elevated telophase index may reflect a delay in completing the mitotic cycle (Rangaswamy, Shanthamurthy, and Arekal 1981).

Analysis of the current findings also revealed the statistically significant increase in DNA fragmentation in *A. cepa* root tip cells at all SFX concentrations and treatment times applied in the comet test is in correlation with the levels of increased CAs as a result of SFX treatment (Figures 4, 5 and 6). CAs involve either structural changes of chromosomes or the number of chromosomes. Joining of chromosomal ends due to the absence of telomere leads to the formation of sticky ends (Nefic et al. 2013; Urgut et al. 2016; Sabeen et al. 2020; Pantano et al. 2021). Stickiness also occurs due to cross‐linking of DNA–DNA or DNA–protein (Barbério, Voltolini, and Mello 2011; Cildir and Liman 2020). Chromosomal laggards and disturbed ana‐telophase are formed when chromosomes do not attach to spindle fibers during cell division (Khanna and Sharma 2013; Tzima, Banti, and Hadjikakou 2022). On the other hand, anaphase chromosome bridge is formed due to dicentric chromosomes resulting from telomere‐to‐telomere fusion or failure of DNA replication enzymes leading to replication stress in common fragile sites (CFSs) such as late replicating or hard‐to‐replicate regions on DNA (Fernandes, Mazzeo, and Marin‐Morales 2007; Finardi, Massari, and Visintin 2020; Ciğerci et al. 2023). Malathion (MT), an organophosphate insecticide, acts as an acetylcholinesterase inhibitor, and showed cytotoxic and genotoxic effects as evaluated by *A. cepa* assay. At the 0.5 mg/mL concentration of MT, anaphase chromosome bridges were observed. Similarly, the reduction in MI was increased from 0.5 to 1.0 mg/mL MT concentrations (Ghisi et al. 2021). Contrary to our findings, SFX did not induce toxic effects on survival, mortality, sex ratio, or body size of *Osmia bicornis* (Schwarz et al. 2022). SFX insecticide has also showed cytotoxic, genotoxic, and apoptotic potential in human blood lymphocytesˈ cell cultures and, thus, raise concerns about health and safety (Sınacı et al. 2023). However, SFX showed less toxic effects compared with imidacloprid and clothianidin which are recently banned neonicotinoids, but more toxic than acetamiprid and thiacloprid in three bee species (Azpiazu et al. 2021). Even sub‐lethal toxic effects of SFX on the red imported fire ant, *Solenopsis invicta* , have been observed (Fengxiang, Yongyue, and Lei 2017). Clothianidin, a neonicotinoid insecticide, induces single‐strand breaks, increases comet tail intensity and length, triggers oxidative stress, concentration‐dependent DNA damage, and increases micronucleus frequency in human lung cells, mouse bone marrow cells, and human blood lymphocytes (Atlı Şekeroğlu et al. 2020; Calderón‐Segura et al. 2012; Calderón‐SeguraM et al. 2015).

Dose‐ and time‐dependent DNA damage was observed in the current study, after the administration of various concentrations of SFX (Figure 6). Human blood lymphocytes exposed to clothianidin for 2 h showed significant, concentration‐dependent increases in DNA damage, measured by comet assay tail length and frequency, across concentrations from 1.2 × 10^−1^ M to 9.5 × 10^−1^ M (Calderón‐Segura et al. 2012). DNA damage is induced when a chemical induces the formation of free radicals and reactive oxygen species, due to which antioxidant processes do not work properly (Rucińska, Sobkowiak, and Gwóźdź 2004; Yıldız et al. 2009). Chronic exposure to triflumezopyrim in earthworms caused oxidative stress, DNA damage, and altered antioxidant enzyme activity (Wen et al. 2021). Exposure to Nitenpyram in human bone marrow increased ROS and SOD, causing DNA damage at lower concentrations (12.5–100 μg/mL), transcriptomic analysis revealed 468 differentially expressed genes, enriching 22 pathways, including cancer‐related pathways (Liu et al. 2022).

SFX has a unique molecular scaffold due to the sulfoximine group in its structure. It has been reported that the insecticide imidacloprid, which molecularly resembles SFX, induced primary DNA damage in the comet test and increased micronucleus and other nuclear abnormalities at high concentrations in the fish *Oreochromis niloticus* , which showed consistency with our results (Rodríguez et al. 2015). Thiamethoxam exposure affected Chinese rare minnows, both sexes exhibited decreased body length and plasma T4 levels, while histological liver damage and delayed gonadal development were observed at a concentration of 50 μg/L, which may disrupt the endocrine system in Chinese rare minnows (Zhu et al. 2019).

Docking studies revealed that SFX binds to DNA with a preference for minor groove recognition, exhibiting a mean binding free energy (Δ*G*) of −5.05 kcal/mol (Table 2, Figures 7 and 8). SFX forms two halogen bonds with DNA bases A3 and T4, along with hydrophobic and hydrogen bond interactions with A3 and G2, as well as a small number of van der Waals contacts with T4, C5, and G6, showing a strong binding affinity (Table 2). On the other hand, the positive control compound MMS showed mixed binding modes, with both minor groove recognition and intercalation, and a mean Δ*G* of −2.94 kcal/mol for intercalation (Table 2). Overall, SFX demonstrated a significantly stronger DNA‐binding interaction compared with MMS, highlighting its potential DNA damage‐inducing capacity. In our opinion, SFX forms stable DNA‐ligand complexes in the DNA minor grooves of the chromatin in the G_1_ phase in *A. cepa* root tip cells, and subsequently, in the S phase (DNA synthesis phase), these stable complexes may cause replication stall in the DNA loci where they are localized. In turn, DNA‐bound SFX (or irreparable DNA damage), which cannot be efficiently removed by the DNA repair system, transforms into DNA double‐strand breaks (DSBs), and in the following mitotic phase, chromosome stickiness and laggard chromosomes that have failed to bind polar microtubules, emerge as a result of DSB‐induced intra‐ and inter‐chromatidic links (Al Achkar, Sabatier, and Dutrillaux 1989; Siri, Martino, and Gottifredi 2021). Since DNA single‐strand breaks can be repaired in a shorter period than double‐strand breaks (Calini, Urani, and Camatini 2002), DNA double‐strand breaks can be expected to be at a higher level in the total DNA damage determined in the comet test, and this deduction is also compatible with SFX's ability to form stable complexes with the DNA minor groove (Figure 8A). Briefly, when docking, CA and comet test data are evaluated together, SFX could be a DNA‐reactive mutagen that has the potential to induce DNA double‐strand break‐type structural damage by forming stable DNA‐ligand complexes.

## 5 Conclusion

According to the *A. cepa* root growth inhibition, ana‐telophase chromosome aberration, comet assay, and computational docking results, it is revealed that SFX causes cytotoxic and genotoxic effects in the root tip cells of the non‐target organism *A. cepa* . DNA docking results provide further explanation at the molecular level for the genotoxic damage caused by SFX and indicate that increased frequency of chromosomal damage such as chromosome laggards, stickiness, and anaphase chromosome bridge may result from SFX forming a more stable complex with the DNA molecule compared with known mutagen MMS. Therefore, in addition to its observed prominent cellular cytotoxicity in *Allium cepa* root meristematic cells, SFX could also be a DNA‐reactive mutagen that exerts a genotoxic effect through its ability to form stable complexes with DNA. Based upon our findings, it is suggested that SFX should be used with caution, along with a minimum recommended rate for the specific crops and pests. It is better that alternatives to SFX should be used to avoid its hazardous effects on environment.

## Author Contributions

**Recep Liman:** conceptualization, investigation, methodology, validation, writing – original draft, formal analysis, supervision. **Muhammad Muddassir Ali:** conceptualization, investigation, writing – original draft, writing – review and editing, methodology, formal analysis. **Erman Salih İstifli:** investigation, software, data curation, formal analysis. **İbrahim Hakkı Ciğerci:** conceptualization, investigation, validation, visualization, supervision, resources. **Ümran Tınaz:** validation, methodology, visualization. **Sidal Kırlangıç:** methodology, validation, visualization, data curation. **Nejla Altay:** methodology, validation, data curation. **Yudum Yeltekin Uğur:** methodology, validation, visualization, data curation.

## Ethics Statement

Ethical approval was waived for this study.

## Consent

Informed consent was obtained from the individuals involved in the study.

## Conflicts of Interest

The authors declare no conflicts of interests.

## Data Availability

The data that support the findings of this study are available from the corresponding author upon reasonable request.

## References

[jemt24807-bib-0001] Al Achkar, W. , L. Sabatier , and B. Dutrillaux . 1989. “How Are Sticky Chromosomes Formed?” Annales de Génétique 32: 10–15. 2751243

[jemt24807-bib-0002] Alaguprathana, M. , M. Poonkothai , M. M. Al‐Ansari , L. Al‐Humaid , and W. Kim . 2022. “Cytogenotoxicity Assessment in *Allium cepa* Roots Exposed to Methyl Orange Treated With Oedogonium Subplagiostomum AP1.” Environmental Research 213: 113612. 35716816 10.1016/j.envres.2022.113612

[jemt24807-bib-0003] Alesawy, M. S. , E. B. Elkaeed , A. A. Alsfouk , A. M. Metwaly , and I. H. Eissa . 2021. “In Silico Screening of Semi‐Synthesized Compounds as Potential Inhibitors for SARS‐CoV‐2 Papain‐Like Protease: Pharmacophoric Features, Molecular Docking, ADMET, Toxicity and DFT Studies.” Molecules 26: 6593. 34771004 10.3390/molecules26216593 PMC8588135

[jemt24807-bib-0004] Ali, M. M. , and İ. H. Ciğerci . 2019. “Genotoxic Evaluation of an Endemic Plant thermopsis turcica Extracts on Liver cancer Cell Line.” Pakistan Journal of Zoology 51: 355–357.

[jemt24807-bib-0005] Ali, M. M. , A. Fatima , S. Nawaz , A. Rehman , M. Javed , and A. Nadeem . 2022a. “Cytotoxic and Genotoxic Evaluation of Bisphenol S on Onion Root Tips by Allium Cepa and Comet Tests.” Environmental Science and Pollution Research 29, no. 59: 88803–88811. 35836054 10.1007/s11356-022-21888-2

[jemt24807-bib-0006] Ali, M. M. , T. Sahar , S. Firyal , et al. 2022b. “Assessment of Cytotoxic, Genotoxic, and Oxidative Stress of Dibutyl Phthalate on Cultured Bovine Peripheral Lymphocytes.” Oxidative Medicine and Cellular Longevity 2022: 1–6. 10.1155/2022/9961513 PMC8970799 35368873

[jemt24807-bib-0007] Alshehri, A. A. , A. M. Almutairi , A. Shafie , N. A. Alshehri , S. M. Almutairi , and F. Anjum . 2023. “"Identification of Potential Inhibitors Targeting DNA Adenine Methyltransferase of *Klebsiella pneumoniae* for Antimicrobial Resistance Management: A Structure‐Based Molecular Docking Study." *Advancements* .” Life Sciences 10, no. 4: 604–608.

[jemt24807-bib-0096] Ambreen, F. , M. A. H. Hashmi , S. Abbas , S. Kouser , F. Latif , and M. Javed , 2023. “Genotoxic Response of Oreochromis niloticus (tilapia) Exposed to Tertiary Mixture of Pesticides.” Advancements in Life Sciences 9, no. 4: 384–390. 10.62940/als.v9i4.1251.

[jemt24807-bib-0010] Atlı Şekeroğlu, Z. , V. Şekeroğlu , B. Aydın , S. Kontaş Yedier , and E. Ilkun . 2020. “Clothianidin Induces DNA Damage and Oxidative Stress in Bronchial Epithelial Cells.” Environmental and Molecular Mutagenesis 61, no. 6: 647–655. 32285515 10.1002/em.22376

[jemt24807-bib-0011] Aydın, G. , and R. Liman . 2020. “Cyto‐Genotoxic Effects of Pinoxaden on *Allium cepa* L. Roots.” Journal of Applied Genetics 61, no. 3: 349–357. 32399682 10.1007/s13353-020-00560-w

[jemt24807-bib-0012] Ayhan, B. S. , T. K. Macar , O. Macar , E. Yalçin , K. Çavuşoğlu , and B. Özkan . 2024. “A Comprehensive Analysis of Royal Jelly Protection Against Cypermethrin‐Induced Toxicity in the Model Organism *Allium cepa* L., Employing Spectral Shift and Molecular Docking Approaches.” Pesticide Biochemistry and Physiology 203: 105997. 39084771 10.1016/j.pestbp.2024.105997

[jemt24807-bib-0013] Azpiazu, C. , J. Bosch , L. Bortolotti , et al. 2021. “Toxicity of the Insecticide Sulfoxaflor Alone and in Combination With the Fungicide Fluxapyroxad in Three Bee Species.” Scientific Reports 6821: 2045–2322. 10.1038/s41598-021-86036-1 PMC7994444 33767274

[jemt24807-bib-0014] Babcock, J. M. , C. B. Gerwick , J. X. Huang , et al. 2011. “Biological Characterization of Sulfoxaflor, a Novel Insecticide.” Pest Management Science 67, no. 3: 328–334. 21308958 10.1002/ps.2069

[jemt24807-bib-0015] Bakadir, K. , A. Kassale , K. Barouni , R. Lakhmiri , and A. Albourine . 2016. “Retention of a Compound of Herbicides, 2, 4‐Dichlorophenoxy Acetic Acid, to a Soil in the Absence and in the Presence of cu (II) and Zn (II) Cations.” Journal of Materials and Environmental Science 7: 1056–1063.

[jemt24807-bib-0016] Banti, C. N. , and S. K. Hadjikakou . 2019. “Evaluation of Genotoxicity by Micronucleus Assay In Vitro and by *Allium cepa* Test In Vivo.” Bio‐Protocol 9, no. 14: e3311. 33654820 10.21769/BioProtoc.3311 PMC7854110

[jemt24807-bib-0017] Barbério, A. , J. C. Voltolini , and M. L. S. Mello . 2011. “Standardization of Bulb and Root Sample Sizes for the *Allium cepa* Test.” Ecotoxicology 20, no. 4: 927–935. 21298340 10.1007/s10646-011-0602-8

[jemt24807-bib-0019] Bhat, S. A. , J. Singh , and A. P. Vig . 2015. “Vermistabilization of Sugar Beet ( *Beta vulgaris* L) Waste Produced From Sugar Factory Using Earthworm *Eisenia fetida* : Genotoxic Assessment by *Allium cepa* Test.” Environmental Science and Pollution Research 22: 11236–11254. 25794577 10.1007/s11356-015-4302-4

[jemt24807-bib-0020] Biovia, D. S. 2016. Discovery Studio. Dassault Systèmes BIOVIA.

[jemt24807-bib-0021] Bonciu, E. , P. Firbas , C. S. Fontanetti , et al. 2018. “An Evaluation for the Standardization of the *Allium cepa* Test as Cytotoxicity and Genotoxicity Assay.” Caryologia 71, no. 3: 191–209.

[jemt24807-bib-0022] Bouhadi, M. , O. Abchir , I. Yamari , et al. 2024. “Genotoxic Effects and Mitosis Aberrations of Chromium (VI) on Root Cells of Vicia faba and Its Molecular Docking Analysis.” Plant Physiology and Biochemistry 207: 108361. 38237423 10.1016/j.plaphy.2024.108361

[jemt24807-bib-0023] Cabuga, C. C., Jr. 2017. “ *Allium cepa* Test: An Evaluation of Genotoxicity.” Proc Int Acad Ecol Environ Sci 7: 12.

[jemt24807-bib-0024] Calderón‐Segura, M. E. , S. Gómez‐Arroyo , R. Villalobos‐Pietrini , et al. 2012. “Evaluation of Genotoxic and Cytotoxic Effects in Human Peripheral Blood Lymphocytes Exposed In Vitro to Neonicotinoid Insecticides News.” Journal of Toxicology 2012, no. 1: 612647. 22545045 10.1155/2012/612647 PMC3321573

[jemt24807-bib-0025] Calderón‐SeguraM, R. , T. C. M. BritoM , M. Calderón‐Ezquerro , and S. Gómez‐Arroyo . 2015. “Genotoxicity of the Neonicotinoid Insecticide Poncho (Clothianidin) on CD1 Mice Based on Alkaline Comet and Micronucleus Assays.” Toxicity and Hazard of Agrochemicals 113: 61174.

[jemt24807-bib-0026] Calini, V. , C. Urani , and M. Camatini . 2002. “Comet Assay Evaluation of DNA Single‐and Double‐Strand Breaks Induction and Repair in C3H10T1/2 Cells.” Cell Biology and Toxicology 18: 369–379. 12484548 10.1023/a:1020811522100

[jemt24807-bib-0027] Cheng, Y. , Y. Bu , L. Tan , et al. 2018. “A Semi‐Field Study to Evaluate Effects of Sulfoxaflor on Honey Bee ( *Apis mellifera* ).” Bull. Insectol 71: 225–233.

[jemt24807-bib-0028] Chung, H. S. , A. M. Abd El‐Aty , S. W. Kim , et al. 2017. “Simultaneous Determination of Sulfoxaflor and Its Metabolites, X11719474 and X11721061, in Brown Rice and Rice Straw After Field Application Using LC‐MS/MS.” International Journal of Environmental Analytical Chemistry 97, no. 2: 99–111.

[jemt24807-bib-0030] Ciğerci, İ. H. , R. Liman , E. S. İstifli , et al. 2023. “Cyto‐Genotoxic and Behavioral Effects of Flubendiamide in *Allium cepa* Root Cells, Drosophila Melanogaster and Molecular Docking Studies.” International Journal of Molecular Sciences 24, no. 2: 1565. 36675079 10.3390/ijms24021565 PMC9861014

[jemt24807-bib-0031] Cildir, D. S. , and R. Liman . 2020. “Cytogenetic and Genotoxic Assessment in *Allium cepa* Exposed to Imazalil Fungicide.” Environmental Science and Pollution Research 27: 20335–20343. 32242316 10.1007/s11356-020-08553-2

[jemt24807-bib-0032] Daina, A. , O. Michielin , and V. Zoete . 2017. “SwissADME: A Free Web Tool to Evaluate Pharmacokinetics, Drug‐Likeness and Medicinal Chemistry Friendliness of Small Molecules.” Scientific Reports 7: 1–13. 28256516 10.1038/srep42717 PMC5335600

[jemt24807-bib-0033] Eberhardt, J. , D. Santos‐Martins , A. Tillack , and S. Forli . 2021. AutoDock Vina 1.2. 0: new docking methods, expanded force field, and Python bindings. 10.1021/acs.jcim.1c00203 PMC10683950 34278794

[jemt24807-bib-0034] EFSA (European Food Safety Authority) . 2014. “Conclusion on the Peer Review of the Pesticide Risk Assessment of the Active Substance Sulfoxaflor.” EFSA Journal 12, no. 5: 170. 10.2903/j.efsa.2009.1415 PMC11926639 40123697

[jemt24807-bib-0035] El‐Ghamery, A. A. , A. I. El‐Nahas , and M. M. Mansour . 2000. “The Action of Atrazine Herbicide as an Inhibitor of Cell Division on Chromosomes and Nucleic Acids Content in Root Meristems of Allium Cepa and *Vicia faba* .” Cytologia 65 3: 277–287.

[jemt24807-bib-0036] Fengxiang, P. , L. Yongyue , and W. Lei . 2017. “Toxicity and Sublethal Effects of Sulfoxaflor on the Red Imported Fire Ant, *Solenopsis invicta* , Ecotoxicology and Environmental Safety.” 139: 147. 10.1016/j.ecoenv.2017.02.014 28189779

[jemt24807-bib-0037] Fernandes, T. C. , D. E. C. Mazzeo , and M. A. Marin‐Morales . 2007. “Mechanism of Micronuclei Formation in Polyploidizated Cells of *Allium cepa* Exposed to Trifluralin Herbicide.” Pesticide Biochemistry and Physiology 88, no. 3: 252–259.

[jemt24807-bib-0038] Finardi, A. , L. F. Massari , and R. Visintin . 2020. “Anaphase Bridges: Not all Natural Fibers Are Healthy.” Genes 11, no. 8: 902. 32784550 10.3390/genes11080902 PMC7464157

[jemt24807-bib-0039] Fusconi, A. , O. Repetto , E. Bona , et al. 2006. “Effect of Cadmium on Meristem Activity and Nucleus Ploidy in Roots of *Pisum sativum* L. cv.” Frisson Seedlings. Environ Exp Bot 58: 253–260.

[jemt24807-bib-0040] Ghisi, N. C. , V. B. Silva , A. A. Roque , and E. C. Oliveira . 2021. “Integrative Analysis in Toxicological Assessment of the Insecticide Malathion in *Allium cepa* L. System.” Brazilian Journal of Biology 83: e240118. 10.1590/1519-6984.240118 34133488

[jemt24807-bib-0041] Gilad, Y. , and H. Senderowitz . 2014. “Docking Studies on DNA Intercalators.” Journal of Chemical Information and Modeling 54: 96–107. 24303988 10.1021/ci400352t

[jemt24807-bib-0042] Glei, M. , T. Schneider , and W. Schlormann . 2016. “Comet Assay: An Essential Tool in Toxicological Research.” Archives of Toxicology 90: 2315–2336. 27378090 10.1007/s00204-016-1767-y

[jemt24807-bib-0043] Gupta, K. , K. Mishra , S. Srivastava , and A. Kumar . 2018. “Cytotoxic Assessment of Chromium and Arsenic Using Chromosomal Behavior of Root Meristem in *Allium cepa* L.” Bulletin of Environmental Contamination and Toxicology 100: 803–808. 29704021 10.1007/s00128-018-2344-2

[jemt24807-bib-0044] Hanwell, M. D. , D. E. Curtis , D. C. Lonie , T. Vandermeersch , E. Zurek , and G. R. Hutchison . 2012. “Avogadro: An Open‐Source Molecular Builder and Visualization Tool.” Journal of Cheminformatics 4: 17. 22889332 10.1186/1758-2946-4-17 PMC3542060

[jemt24807-bib-0045] Ihara, M. , and K. Matsuda . 2018. “Neonicotinoids: Molecular Mechanisms of Action, Insights Into Resistance and Impact on Pollinators.” Current Opinion in Insect Science 2018, no. 30: 86–92. 10.1016/j.cois.2018.09.009 30553491

[jemt24807-bib-0046] Karaismailoğlu, C. 2022. “Cytotoxic and Genotoxic Influences of Oxyfluorfen on the Somatic Cells of *Allium cepa* .” Kahramanmaraş Sütçü İmam Üniversitesi Tarım Ve Doğa Dergisi 25, no. 2: 207–214.

[jemt24807-bib-0047] Khanna, N. , and S. Sharma . 2013. “ *Allium cepa* Root Chromosomal Aberration Assay: A Review.” Indian Journal of Pharmaceutical and Biological Research 1: 105–119.

[jemt24807-bib-0048] Kocyigit, A. , H. Keles , S. Selek , S. Guzel , H. Celik , and O. Erel . 2005. “Increased DNA Damage and Oxidative Stress in Patients With Cutaneous Leishmaniasis.” Mutat Res‐Gen Tox En 585, no. 1: 71–78. 10.1016/j.mrgentox.2005.04.012 16005255

[jemt24807-bib-0050] Kyriakopoulou, K. , I. Kandris , I. Pachiti , et al. 2017. “Collection and Analysis of Pesticide Residue Data for Pollen and Nectar – Final Report.” EFSA Supporting Publications 14: 1–96.

[jemt24807-bib-0051] LeBaron, M. J. , D. R. Geter , R. J. Rasoulpour , et al. 2013. “An Integrated Approach for Prospectively Investigating a Mode‐Of‐Action for Rodent Liver Effects.” Toxicology and Applied Pharmacology 270, no. 2: 164–173. 23607986 10.1016/j.taap.2013.04.009

[jemt24807-bib-0052] Liman, R. , M. M. Ali , İ. H. Ciğerci , E. S. İstifli , and C. Sarıkurkcu . 2021. “Cytotoxic and Genotoxic Evaluation of Copper Oxychloride Through Allium Test and Molecular Docking Studies.” Environmental Science and Pollution Research 28, no. 33: 44998–45008. 33860424 10.1007/s11356-021-13897-4

[jemt24807-bib-0053] Liman, R. , M. M. Ali , E. S. Istifli , İ. H. Ciğerci , and E. Bonciu . 2022. “Genotoxic and Cytotoxic Effects of Pethoxamid Herbicide on *Allium cepa* Cells and Its Molecular Docking Studies to Unravel Genotoxicity Mechanism.” Environmental Science and Pollution Research 29: 63127–63140. 35449332 10.1007/s11356-022-20166-5

[jemt24807-bib-0054] Liman, R. , and S. Ozkan . 2019. “Cytotoxicity and Genotoxicity in *Allium cepa* L. Root Meristem Cells Exposed to the Herbicide Penoxsulam. *Celal Bayar University Journal of* .” Science 15, no. 2: 221–226.

[jemt24807-bib-0055] Liu, W. , Z. Li , X. Cui , et al. 2022. “Genotoxicity, Oxidative Stress and Transcriptomic Effects of Nitenpyram on Human Bone Marrow Mesenchymal Stem Cells.” Toxicology and Applied Pharmacology 446: 116065. 35568224 10.1016/j.taap.2022.116065

[jemt24807-bib-0056] Ma, K. , Q. Tang , B. Zhang , P. Liang , B. Wang , and X. Gao . 2019. “Overexpression of Multiple Cytochrome P450 Genes Associated With Sulfoxaflor Resistance in *Aphis gossypii* Glover.” Pesticide Biochemistry and Physiology 157: 204–210. 31153470 10.1016/j.pestbp.2019.03.021

[jemt24807-bib-0058] Moller, P. 2018. “The Comet Assay: Ready for 30 More Years.” Mutagenesis 33: 1–7. 29325088 10.1093/mutage/gex046

[jemt24807-bib-0059] Morris, G. M. , R. Huey , W. Lindstrom , et al. 2009. “AutoDock4 and AutoDockTools4: Automated Docking With Selective Receptor Flexibility.” Journal of Computational Chemistry 30: 2785–2791. 19399780 10.1002/jcc.21256 PMC2760638

[jemt24807-bib-0060] Nefic, H. , J. Musanovic , A. Metovic , and K. Kurteshi . 2013. “Chromosomal and Nuclear Alterations in Root Tip Cells of *Allium cepa* L.” Induced by Alprazolam. Medical Archives 67, no. 6: 388. 25568504 10.5455/medarh.2013.67.388-392 PMC4272485

[jemt24807-bib-0061] Pantano, G. , D. Mazzeo , S. Th , M. Morales , P. Fadini , and A. Mozeto . 2021. “Toxicity of the Sawdust Used for Phosphorus Recovery in Aeutrophic Reservoir: Experiments With Lactuca Sativa and *Allium cepa* .” Environmental Science and Pollution Research 28: 18276–18283. 33410013 10.1007/s11356-020-11868-9

[jemt24807-bib-0063] Pellegri, V. , G. Gorbi , and A. Buschini . 2020. “DNA Damage Detection by Comet Assay on *Daphnia magna* : Application in Freshwater Biomonitoring.” Sci Total Environ 705: 135780. 31972938 10.1016/j.scitotenv.2019.135780

[jemt24807-bib-0064] Rangaswamy, V. , K. B. Shanthamurthy , and G. D. Arekal . 1981. “Cytological Effects of Industrial Effluent on Somatic Cells of *Allium cepa* .” In Perspective of Cytology and Genetics, edited by G. K. Manna and V. Sinha , 303–308. Delhi: Hind Asia Publication.

[jemt24807-bib-0065] Rank, J. , and M. H. Nielsen . 1994. “Evaluation of the Allium Anaphase‐Telophase Test in Relation to Genotoxicity Screening of Industrial Wastewater.” Mutation Research, Environmental Mutagenesis and Related Subjects 312, no. 1: 17–24. 10.1016/0165-1161(94)90004-3 7507212

[jemt24807-bib-0066] Ricci, C. G. , and P. A. Netz . 2009. “Docking Studies on DNA‐Ligand Interactions: Building and Application of a Protocol to Identify the Binding Mode.” Journal of Chemical Information and Modeling 49: 1925–1935. 19655805 10.1021/ci9001537

[jemt24807-bib-0067] Rodríguez, Y. A. , C. A. Christofoletti , J. Pedro , et al. 2015. “Allium Cepa and *Tradescantia pallida* Bioassays to Evaluate Effects of the Insecticide Imidacloprid.” Chemosphere 120: 438–442. 25225953 10.1016/j.chemosphere.2014.08.022

[jemt24807-bib-0068] Rucińska, R. E. N. A. T. A. , R. O. B. E. R. T. Sobkowiak , and E. A. Gwóźdź . 2004. “Genotoxicity of Lead in Lupin Root Cells as Evaluated by the Comet Assay.” Cellular & Molecular Biology Letters 9, no. 3: 519–528. 15332128

[jemt24807-bib-0069] Sabeen, M. , Q. Mahmood , Z. A. Bhatti , et al. 2020. “ *Allium cepa* Assay Based Comparative Study of Selected Vegetables and the Chromosomal Aberrations due to Heavy Metal Accumulation.” Saudi Journal of Biological Sciences 27, no. 5: 1368–1374. 32346347 10.1016/j.sjbs.2019.12.011 PMC7182997

[jemt24807-bib-0070] Sarkar, A. , S. Concilio , L. Sessa , F. Marrafino , and S. Piotto . 2024. “Advancements and Novel Approaches in Modified Autodock Vina Algorithms for Enhanced Molecular Docking. Results.” Chemistry 101319: 101319.

[jemt24807-bib-0071] Schwarz, J. M. , A. C. Knauer , M. J. Allan , et al. 2022. “No Evidence for Impaired Solitary Bee Fitness Following Pre‐Flowering Sulfoxaflor Application Alone or in Combination With a Common Fungicide in a Semi‐Field Experiment.” Environment International 164: 107252. 35483184 10.1016/j.envint.2022.107252

[jemt24807-bib-0072] Sheikh, N. , H. Patowary , and R. A. Laskar . 2020. “Screening of Cytotoxic and Genotoxic Potency of Two Pesticides (Malathion and Cypermethrin) on *Allium cepa* L.” Cell. Toxicol 16: 291–299. 10.1007/s13273-020-00077-7.

[jemt24807-bib-0073] Silveira, G. L. , M. G. F. Lima , G. B. dos Reis , M. J. Palmieri , and L. F. Andrade‐Vieria . 2017. “Toxic effects of environmental pollutants: Comparative investigation using Allium cepa L. and Lactuca sativa L.” In Toxic Effects of Environmental Pollutants: Comparative Investigation Using *Allium cepa* L Chemosphere 178: 359–367. 10.1016/j.chemosphere.2017.03.048 28340458

[jemt24807-bib-0074] Sınacı, C. , A. Çelik , D. Yetkin , S. Çevik , and G. Güler . 2023. “Sulfoxaflor Insecticide Exhibits Cytotoxic or Genotoxic and Apoptotic Potential via Oxidative Stress‐Associated DNA Damage in Human Blood Lymphocytes Cell Cultures.” Drug Chem Toxicol 46: 972–983. 36036091 10.1080/01480545.2022.2114006

[jemt24807-bib-0075] Singh, D. , and B. K. Roy . 2017. “Evaluation of Malathion‐Induced Cytogenetical Effects and Oxidative Stress in Plants Using Allium Test.” Acta Physiologiae Plantarum 39: 1–10.

[jemt24807-bib-0076] Siri, S. O. , J. Martino , and V. Gottifredi . 2021. “Structural Chromosome Instability: Types, Origins, Consequences, and Therapeutic Opportunities.” Cancers 13: 3056. 34205328 10.3390/cancers13123056 PMC8234978

[jemt24807-bib-0077] Siviter, H. , M. J. F. Brown , and E. Leadbeater . 2018. “Sulfoxaflor Exposure Reduces Bumblebee Reproductive Success.” Nature 2018, no. 561: 108–111. 10.1038/s41586-018-0430-6 30111837

[jemt24807-bib-0078] Siviter, H. , J. Horner , M. J. Brown , and E. Leadbeater . 2020. “Sulfoxaflor Exposure Reduces Egg Laying in Bumblebees *Bombus terrestris* .” Journal of Applied Ecology 57, no. 1: 160–169. 32055075 10.1111/1365-2664.13519 PMC7004077

[jemt24807-bib-0079] Skouras, P. J. , E. Karanastasi , V. Demopoulos , M. Mprokaki , G. J. Stathas , and J. T. Margaritopoulos . 2023. “Toxicity and Influence of Sublethal Exposure to Sulfoxaflor on the Aphidophagous Predator *Hippodamia variegata* (Coleoptera: Coccinellidae).” Toxics 11, no. 6: 533. 37368633 10.3390/toxics11060533 PMC10305232

[jemt24807-bib-0080] Soliman, M. I. , and G. T. Ghoneam . 2004. “The Mutagenic Potentialities of Some Herbicides Using *Vicia faba* as a Biological System.” Biotechnology 3: 140–154.

[jemt24807-bib-0081] Sparks, T. C. , G. B. Watson , M. R. Loso , C. Geng , J. M. Babcock , and J. D. Thomas . 2013. “Sulfoxaflor and the Sulfoximine Insecticides: Chemistry, Mode of Action and Basis for Efficacy on Resistant Insects.” Pesticide Biochemistry and Physiology 107, no. 1: 1–7. 25149228 10.1016/j.pestbp.2013.05.014

[jemt24807-bib-0082] Torres, P. H. , A. C. Sodero , P. Jofily , and F. P. Silva Jr. 2019. “Key Topics in Molecular Docking for Drug Design.” International Journal of Molecular Sciences 20, no. 18: 45–74. 10.3390/ijms20184574 PMC6769580 31540192

[jemt24807-bib-0083] Turan, I. , D. Canbolat , S. Demir , et al. 2023. “An Investigation of the Protective Effect of *Rhododendron luteum* Extract on Cisplatin‐Induced DNA Damage and Nephrotoxicity, and Biochemical Parameters in Rats.” Pakistan Veterinary Journal 43, no. 3: 442–448. 10.29261/pakvetj/2023.047.

[jemt24807-bib-0084] Tzima, C. S. , C. N. Banti , and S. K. Hadjikakou . 2022. “Assessment of the Biological Effect of Metal Ions and Their Complexes Using Allium Cepa and *Artemia salina* Assays: A Possible Environmental Implementation of Biological Inorganic Chemistry.” JBIC Journal of Biological Inorganic Chemistry 27, no. 7: 611–629. 36149503 10.1007/s00775-022-01963-2 PMC9569305

[jemt24807-bib-0085] Urgut, O. S. , I. I. Ozturk , C. N. Banti , et al. 2016. “New Antimony (III) Halide Complexes With Dithiocarbamate Ligands Derived From Thiuram Degradation: The Effect of the molecule's Close Contacts on In Vitro Cytotoxic Activity.” Materials Science and Engineering: C 58: 396–408. 26478326 10.1016/j.msec.2015.08.030

[jemt24807-bib-0086] Wang, G. , and W. Zhu . 2016. “Molecular Docking for Drug Discovery and Development: A Widely Used Approach but From Perfect.” Future Sci 8, no. 14: 1707–1710. 10.4155/fmc-2016-0143 27578269

[jemt24807-bib-0087] Watson, G. B. , M. W. Siebert , N. X. Wang , M. R. Loso , and T. C. Sparks . 2021. “Sulfoxaflor–A Sulfoximine Insecticide: Review and Analysis of Mode of Action, Resistance and Cross‐Resistance.” Pesticide Biochemistry and Physiology 178: 104924. 34446200 10.1016/j.pestbp.2021.104924

[jemt24807-bib-0088] Wen, S. , C. Liu , Y. Wang , et al. 2021. “Oxidative Stress and DNA Damage in Earthworm ( *Eisenia fetida* ) Induced by Triflumezopyrim Exposure.” Chemosphere 264: 128499. 33049500 10.1016/j.chemosphere.2020.128499

[jemt24807-bib-0089] Yıldız, M. , İ. H. Ciğerci , M. Konuk , A. F. Fidan , and H. Terzi . 2009. “Determination of Genotoxic Effects of Copper Sulphate and Cobalt Chloride in *Allium cepa* Root Cells by Chromosome Aberration and Comet Assays.” Chemosphere 75, no. 7: 934–938. 19201446 10.1016/j.chemosphere.2009.01.023

[jemt24807-bib-0090] Zhang, X. , X. Wang , Y. Liu , K. Fang , and T. Liu . 2020. “The Toxic Effects of Sulfoxaflor Induced in Earthworms ( *Eisenia fetida* ) Under Effective Concentrations.” International Journal of Environmental Research and Public Health 17, no. 5: 1740. 32155971 10.3390/ijerph17051740 PMC7084856

[jemt24807-bib-0091] Zhang, Y. , G. Zhang , X. Zhou , and Y. Li . 2013. “Determination of Acetamiprid Partial‐Intercalative Binding to DNA by Use of Spectroscopic, Chemometrics, and Molecular Docking Techniques.” Analytical and Bioanalytical Chemistry 405: 8871–8883. 23975088 10.1007/s00216-013-7294-2

[jemt24807-bib-0092] Zhu, L. , W. Li , J. Zha , N. Li , and Z. Wang . 2019. “Chronic Thiamethoxam Exposure Impairs the HPG and HPT Axes in Adult Chinese Rare Minnow ( *Gobiocypris rarus* ): Docking Study, Hormone Levels, Histology, and Transcriptional Responses.” Ecotoxicology and Environmental Safety 185: 109683. 31550567 10.1016/j.ecoenv.2019.109683

[jemt24807-bib-0093] Zhu, Y. , M. R. Loso , and G. B. Watson . 2011. “Discovery and Characterization of Sulfoxaflor, a Novel Insecticide Targeting Sap‐Feeding Pests.” Journal of Agricultural and Food Chemistry 59: 2950–2957. 21105655 10.1021/jf102765x

